# Microstructural data of six recent brachiopod species: SEM, EBSD, morphometric and statistical analyses

**DOI:** 10.1016/j.dib.2018.02.071

**Published:** 2018-03-06

**Authors:** Facheng Ye, Gaia Crippa, Claudio Garbelli, Erika Griesshaber

**Affiliations:** a Dipartimento di Scienze della Terra "A. Desio", Università degli Studi di Milano, Milan, Italy; b State Key Laboratory of Palaeobiology and Stratigraphy, Nanjing Institute of Geology and Palaeontology, Chinese Academy of Sciences, Nanjing, China; c Department für Geo- und Umweltwissenschaften, Ludwig-Maximilians Universitat Munchen, Munich, Germany

## Abstract

Here, we provide the dataset associated with the research article “Mapping of recent brachiopod microstructure: A tool for environmental studies” [1]. We present original data relative to morphometric and statistical analyses performed on the basic shell structural units (the secondary layer fibres) of brachiopod shells belonging to six extant species adapted to different environmental conditions. Based on SEM micrographs of the secondary layer, fibres from ventral and dorsal valves, and from different shell positions, showing regular and symmetrical cross sectional outlines, were chosen for morphometric measurements using Adobe Photoshop CS6, Image-Pro Plus 6.0 and ImageJ. To work out the reliability of the measurements, the most significant parameters were tested for their probability density by distribution plots; for data visualization and dimension reduction, principal component analysis (PCA) was performed using R 3.3.0 [2] and independent-samples *t*-tests were performed using SPSS Statistics (IBM Version 22.0. Armonk, NY). Besides a quantitative analysis, a qualitative description of the shell microstructure is provided by detailed SEM imaging and EBSD measurements.

**Specifications Table**

**Table:** 

Subject area	Structural biology
More specific subject area	Brachiopod shell microstructures
Type of data	Tables and graphs of statistical analyses
	SEM and EBSD images
How data was acquired	SEM: Cambridge S-360 scanning electron microscope with lanthanum hexaboride (LaB6) source and operating at an acceleration voltage of 20 kV
	EBSD: Hitachi SU5000 field emission SEM, equipped with a Nordlys II EBSD detector and AZTec acquisition software
	Morphometric measurements performed with Adobe Photoshop CS6, Image-Pro Plus 6.0 and ImageJ; distribution plots with Excel 2013; principal component analysis (PCA) with R 3.3.0 [2]; independent-sample *t*-tests with SPSS Statistics (IBM Version 22.0. Armonk, NY).
Data format	Analyzed
Experimental factors	Brachiopod shells were embedded in epoxy resin (not all), cut along the longitudinal (or transversal) axis, and immersed in 36 volume hydrogen peroxide (H_2_*O*_2_) for 24 hours to remove organic matter. Sectioned surfaces were smoothed with silicon carbide (SiC) powder, etched with 5% hydrochloric acid (HCl) for 3 seconds, and then rinsed in deionised water and dried [3]. Then they were: 1) gold coated for SEM analysis; 2) mechanically grinded and polished down to a grain size of 1 μm, etch-polished with colloidal alumina (particle size ~ 0.06 μm) in a vibratory polisher and coated with 4–6 nm of carbon for EBSD analysis.
Experimental features	Morphometric measurements and analysis of fibres of the secondary layer based on SEM micrographs, EBSD and statistics (distribution plots, principal component analysis and independent-sample t-tests).
Data source location	Doubtful Sound, New Zealand, 45 °18'00'' S, 166 °58'45'' E
	Kaka Point, New Zealand, 46 °38'66'' S, 169 °78'23'' E
	Trolval Island, Ryder Bay, Antarctica, 67 °35.44' S, 68 °12.44' W
	Signy Island, Antarctica, 60 °43' S, 45 °36' W
	Tuscan Archipelago, Tyrrhenian Sea, Italy, 42 °26' N, 10 °04' E
Data accessibility	Data is with this article

**Value of the data**

• These data provide a quantitative and qualitative description of the microstructure of recent brachiopod shells using several tools: SEM, EBSD, morphometric and statistical analyses.
• These methods may be applied to other invertebrates and to fossil shells to objectively describe and compare their microstructures.
• These data are valuable to researchers investigating invertebrate biomineralization patterns.

## 1 Data

Brachiopod calcite shells are high resolution biomineral archives used to reconstruct global marine environments in the recent and deep past [4], [5], [6], [7], [8], [9], [10]. Biominerals, the hard parts produced by organisms for support and protection, are one of the best tools to use, as they are high-resolution archives of the environmental conditions prevailing during their growth. Here, we focus on the basic structural units (fibres) of the secondary calcite layer of six recent rhynchonelliformean brachiopods. Based on SEM and EBSD analyses, 1197 morphological measurements of the fibres were performed and statistically analyzed, comparing the size and shape of the fibres in different valves of the same specimen, at different positions within the valve, in different shell layer successions, in different species and in different environmental conditions.

## 2 Experimental design, materials and methods

### 2.1 Sample collections

Six extant rhynchonelliformean brachiopod species (21 adult specimens) were chosen for microstructure analyses (Table 1). They have either a two-layer shell sequence or a three-layer shell sequence, both comprising a fibrous secondary layer, and are adapted to different environmental conditions, from Signy and Trolval Islands, Antarctica, to Doubtful Sound and kaka point, New Zealand to the Tuscan Archipelago, Mediterranean Sea.

**Table 1:** Details of the studied materials for shell microstructure analyses. The name of the species, the corresponding ID and museum number, the type of valve and the number of SEM micrographs are shown. The shell succession of each species, the localities of provenance of the specimens and the corresponding geographic coordinates, depth (D), temperature (T) and salinity (S) are also indicated.

Species		ID number		Valve	SEM micrographs number	Shell sequence	Provenance and environmental parameters
Terebratulida	*Liothyrella uva*	LUH1	LUH1 (MPUM 11565)	ventral	40	I, II layers	Trolval Island, Ryder Bay, Antarctica
		LUH2	LUH2 (MPUM 11566)	ventral	28		67° 35.44' S, 68° 12.44' W
		LUH3	LUH3 (MPUM 11567)	ventral	34		T: -2/+2 °C, S: 34 PSU
			LUH3A (MPUM 11591)	dorsal	21		Signy Island (D: 10 m), Antarctica
			LUH3C (MPUM 11591)	dorsal	27		60° 43' S, 45° 36' W
			LUH3P (MPUM 11591)	dorsal	16		T: -2/+2 °C, S: 34 PSU
		LU	LUU (MPUM 11569)	ventral	17		
			LUA (MPUM 11568)	ventral	19		
		LUV/LUD	LUV (MPUM 11560)	ventral	48		
			LUVT (MPUM 11559)	ventral	42		
			LUDCA (MPUM 11592)	dorsal	26		
			LUDP (MPUM 11592)	dorsal	19		
	*Gryphus vitreus*	1D	1DA (MPUM 11595)	ventral	53	I, II, III layers	Tuscan Archipelago (D: 140–160 m between the Island of Pianosa and Montecristo), Tyrrhenian Sea, Italy
			1DB (MPUM 11596)	dorsal	58		42° 26' N, 10° 04' E
		GV	GVV (MPUM 11597)	ventral	34		T: 13–15 °C, S: 39 PSU
			GVD (MPUM 11598)	dorsal	23		
			BO(GVD) (MPUM 11598)	dorsal	24		
		GV3	GV3A (MPUM 11599)	ventral	10		
			GV3C (MPUM 11599)	ventral	12		
			GV3U (MPUM 11599)	ventral	31		
			GV3 (MPUM 11600)	dorsal	15		
		GV4	GV4VA (MPUM 11601)	ventral	12		
			GV4VC1 (MPUM 11601)	ventral	8		
			GV4VC2 (MPUM 11601)	ventral	13		
			GV4VP (MPUM 11601)	ventral	10		
			GV4DA (MPUM 11602)	dorsal	20		
			GV4DC1 (MPUM 11602)	dorsal	20		
			GV4DC2 (MPUM 11602)	dorsal	27		
			GV4DP (MPUM 11602)	dorsal	22		
		GV5	GV5A1	dorsal	2		
			GV5A2	dorsal	12		
	*Liothyrella neozelanica*	1 C	1CA (MPUM 11589)	ventral	62	I, II, III layers	Doubtful Sound (D: 18 m), New Zealand
			1CB (MPUM 11590)	dorsal	82		45° 18' 00'' S, 166° 58' 45'' E
		LZ	LZ (MPUM 11579)	ventral and dorsal	92		T: 11–17 ° C, S: 34.8 PSU
			LZA/LZA1 (MPUM 11580)	ventral and dorsal	45		
			LZA1 (MPUM 11580)	ventral and dorsal	25		
			LZC/LZCC/LZCV (MPUM 11582)	ventral and dorsal	44		
			LZCV (MPUM 11582)	ventral	20		
			LZP/LZP1 (MPUM 11581)	ventral and dorsal	40		
			LZP1 (MPUM 11581)	ventral and dorsal	22		
		LN	LNA (MPUM 11571)	ventral	27		
			LNU (MPUM 11572)	ventral	21		
			LND1 (MPUM 11573)	dorsal	24		
			LND2 (MPUM 11574)	dorsal	28		
			LND3 (MPUM 11575)	dorsal	22		
			LND4 (MPUM 11576)	dorsal	26		
			LND5 (MPUM 11577)	dorsal	18		
			LND6 (MPUM 11578)	dorsal	10		
	*Calloria inconspicua*	1CC	1CC (MPUM 11593)	ventral and dorsal	27	I, II layers	Doubtful Sound (D: 18 m), New Zealand
		CI	CI (MPUM 11594)	ventral and dorsal	43		45° 18' 00'' S, 166° 58' 45'' E
							T: 11–17 °C, S: 34.8 PSU
	*Magasella sanguinea*	TS1	TS1 (MPUM 11603)	ventral and dorsal	61	I, II layers	Doubtful Sound (D: 18 m), New Zealand
			TS1A (MPUM 11604)	ventral and dorsal	24		45° 18' 00'' S, 166° 58' 45'' E
			TS1C (MPUM 11604)	ventral and dorsal	32		T: 11–17 °C °C, S: 34.8 PSU
			TS1P (MPUM 11604)	ventral and dorsal	40		
							
Rhynchonellida	*Notosaria nigricans*	NN	NN (MPUM 11605)	ventral and dorsal	30	I, II layers	Doubtful Sound (D: 18 m), New Zealand
			NN2 (MPUM 11605)	ventral and dorsal	29		45° 18' 00'' S, 166° 58' 45'' E
		NN1	NN1 (MPUM 11606)	ventral and dorsal	34		T: 11-17 °C °C, S: 34.8 PSU
		NN2	NN2VA (MPUM 11607)	ventral	20		Kaka Point (D: 2-15m) New Zealand
			NN2VB (MPUM 11607)	ventral	29		46° 38' 66'' S, 169° 78' 23'' E
			NN2VC (MPUM 11607)	ventral	20		T: 14 °C, S: 34–35 PSU
			NN2DA (MPUM 11608)	dorsal	24		
			NN2DC (MPUM 11608)	dorsal	27		
			NN2DP (MPUM 11608)	dorsal	15		
		NN3	NN3 (MPUM 11609)	ventral and dorsal	47		
		1DC	1DC (MPUM 11610)	ventral	41		

### 2.2 SEM

**Plate 1:**
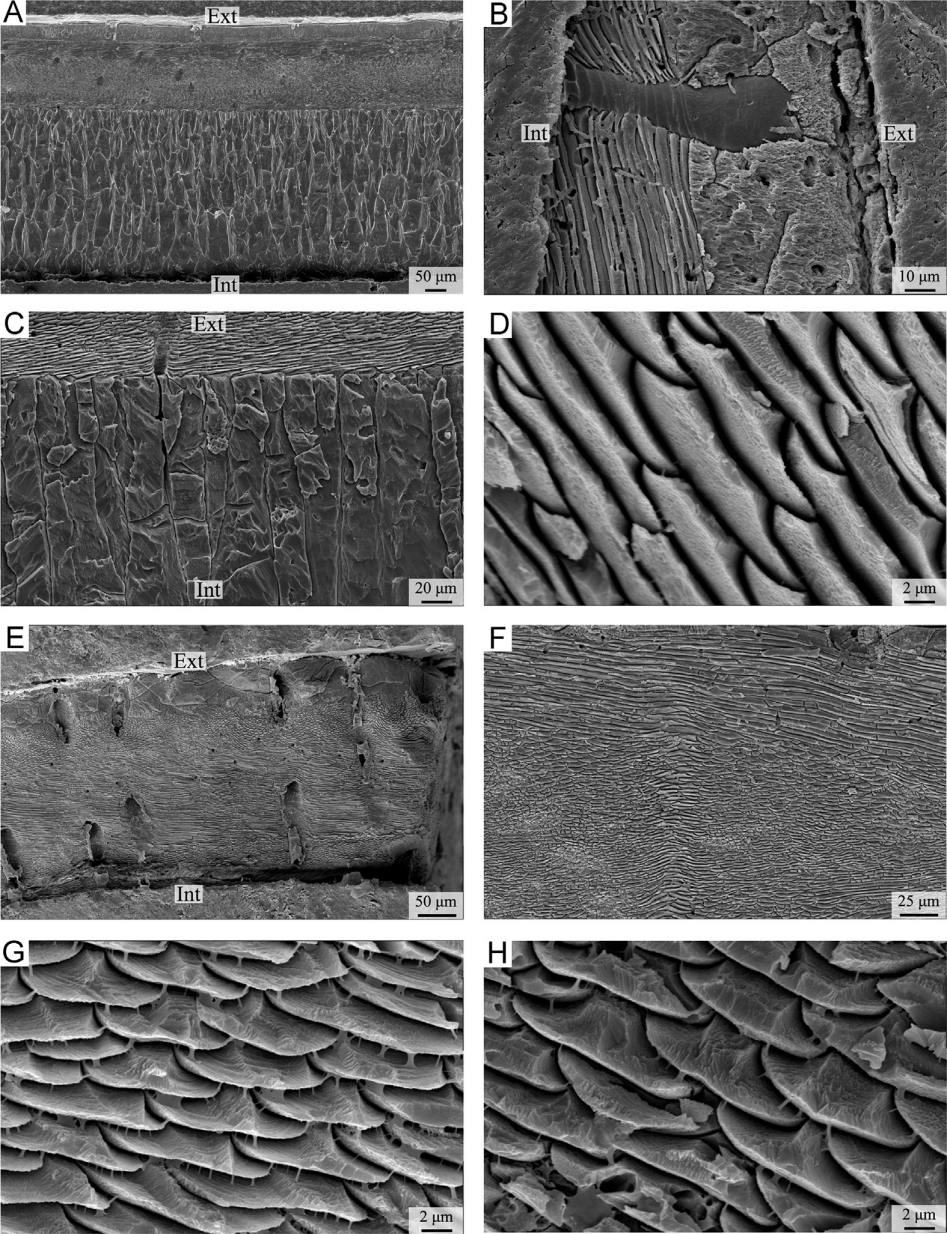
A-D) *Liothyrella neozelanica*. A) complete shell succession from primary to tertiary layer with crossing endopunctae (ventral valve); B) endopuncta crossing the primary and secondary layer (ventral valve); C) transition zone between the secondary and the tertiary layers (dorsal valve); D) enlarged photo showing fibres in transverse section (dorsal valve). E-H) *Liothyrella uva*. E) complete shell succession from primary to secondary layer with crossing endopunctae (ventral valve); F) change in the orientation of fibres within the fibrous secondary layer (parallel, oblique and transverse) (ventral valve); G, H) enlarged photo showing fibres in transverse section (ventral valve). Ext: external part of the shell; Int: internal part of the shell.

**Plate 2:**
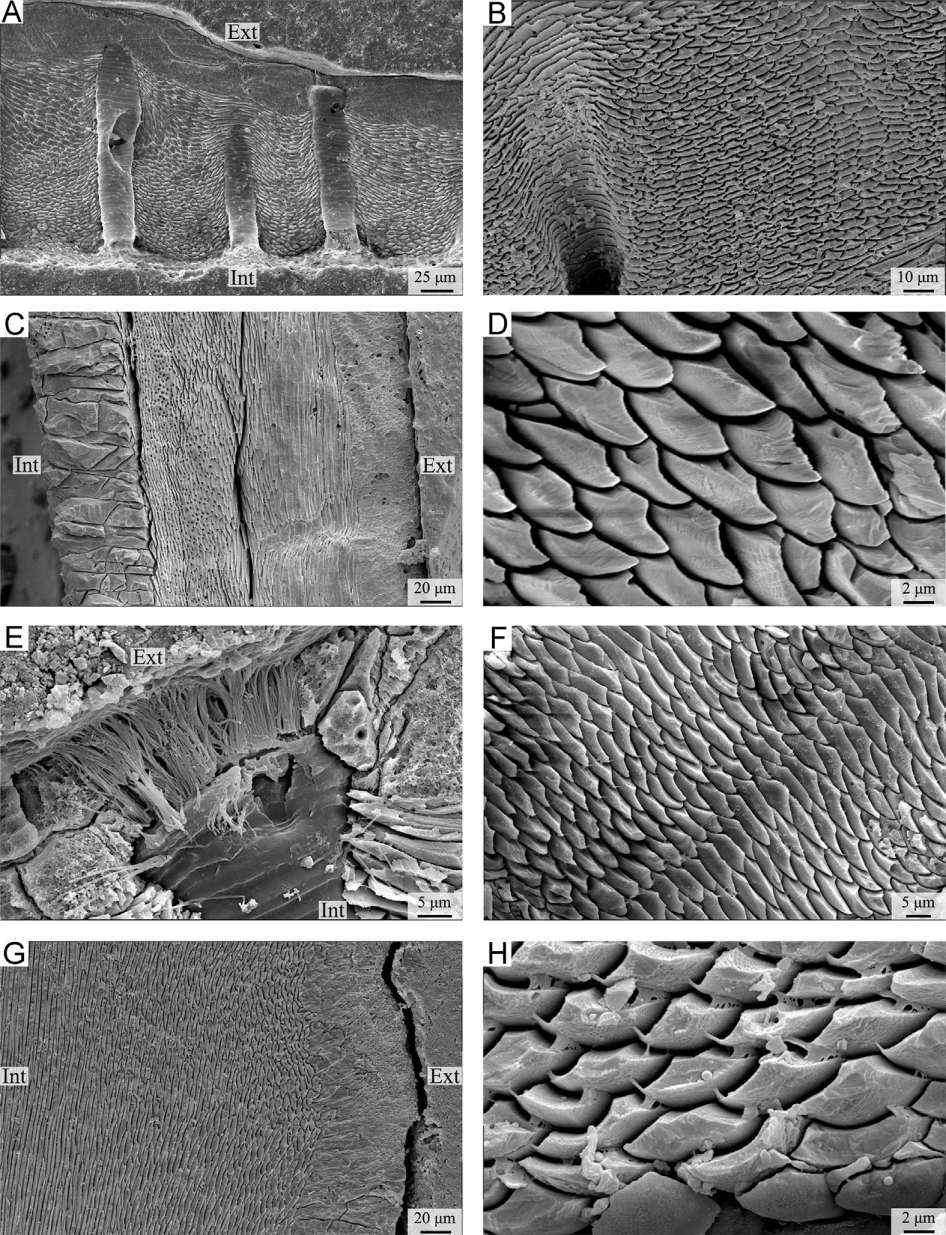
A-B) *Calloria inconspicua*. A) complete shell succession from primary to secondary layer with endopunctae (ventral valve); B) fibrous secondary layer with endopuncta (ventral valve). C-D) *Gryphus vitreus*. C) complete shell succession from primary to tertiary layer (dorsal valve); D) enlarged photo showing fibres in transverse section (dorsal valve). E-F) *Magasella sanguinea*. E) details of an endopuncta (dorsal valve); F) fibrous secondary layer (dorsal valve). G-H) *Notosaria nigricans*. G) primary layer and fibrous secondary layer (dorsal valve); H) details of fibres in the secondary layer (ventral valve). Ext: external part of the shell; Int: internal part of the shell.

**Plate 3:**
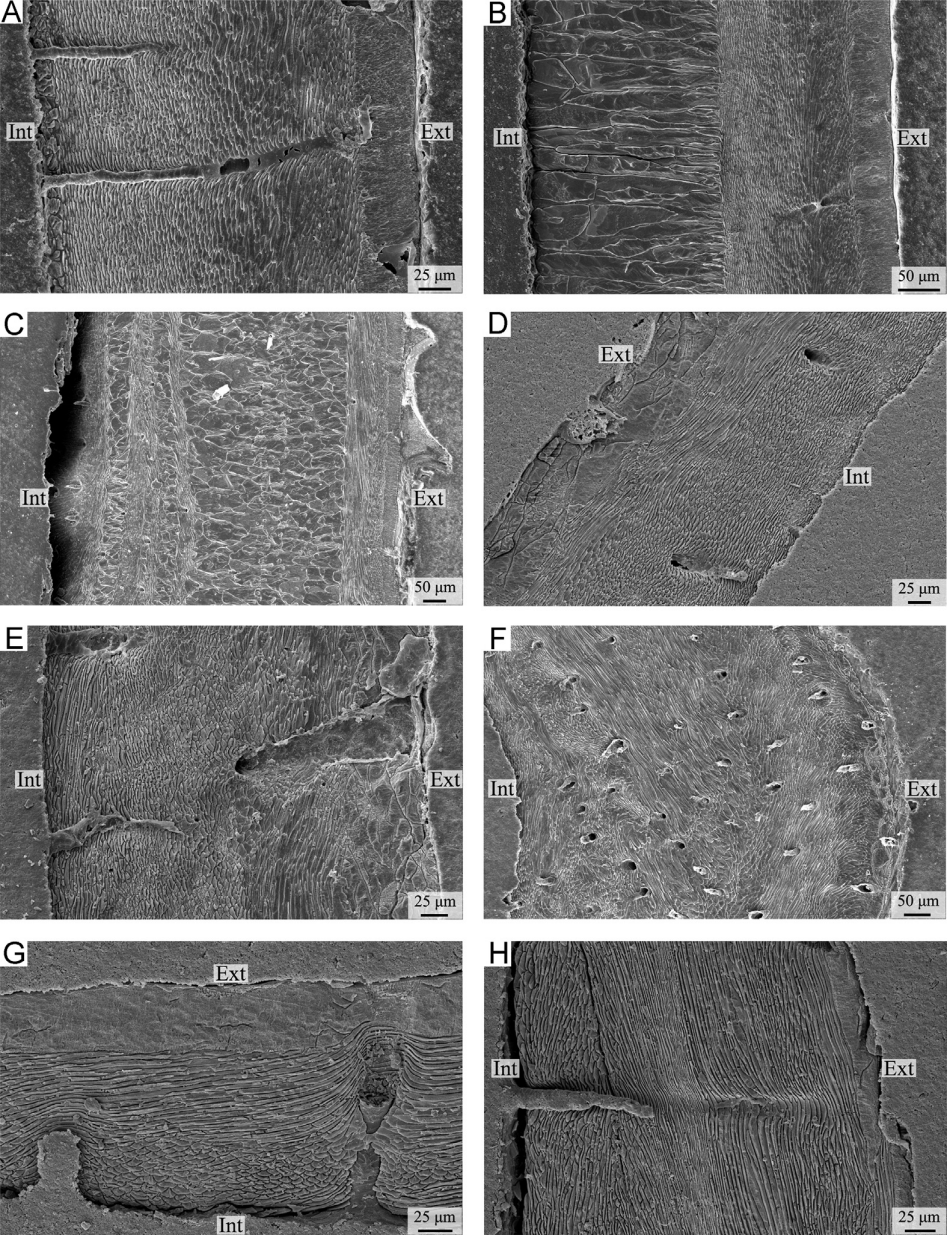
A-C) *Liothyrella neozelanica*. A) complete shell succession showing the change in the orientation of fibres from oblique to transverse from the exterior to the interior of the secondary layer (anterior part, ventral valve, longitudinal section); B) complete shell succession showing the change in the orientation of fibres from oblique to transverse from the exterior to the interior of the secondary layer (central part, ventral valve, longitudinal section); C) complete shell succession showing the change in the orientation of fibres from transverse to oblique from the exterior to the interior of the secondary layer, and the alternations of the secondary and tertiary layers (posterior part, ventral valve, longitudinal section). D-F) *Liothyrella uva*. D-E) complete shell succession showing the change in the orientation of fibres from oblique to transverse from the exterior to the interior of the secondary layer (central part, dorsal valve, longitudinal section); F) complete shell succession showing several sublayers with variable fibre orientation (posterior part, ventral valve, longitudinal section). G-H) *Calloria inconspicua*. G) complete shell succession showing the change in the orientation of fibres from oblique to transverse to oblique from the exterior to the interior of the secondary layer (anterior part, ventral valve, longitudinal section); H) complete shell succession showing several sublayers with variable fibre orientation (posterior part, ventral valve, longitudinal section). Ext: external part of the shell; Int: internal part of the shell.

**Plate 4:**
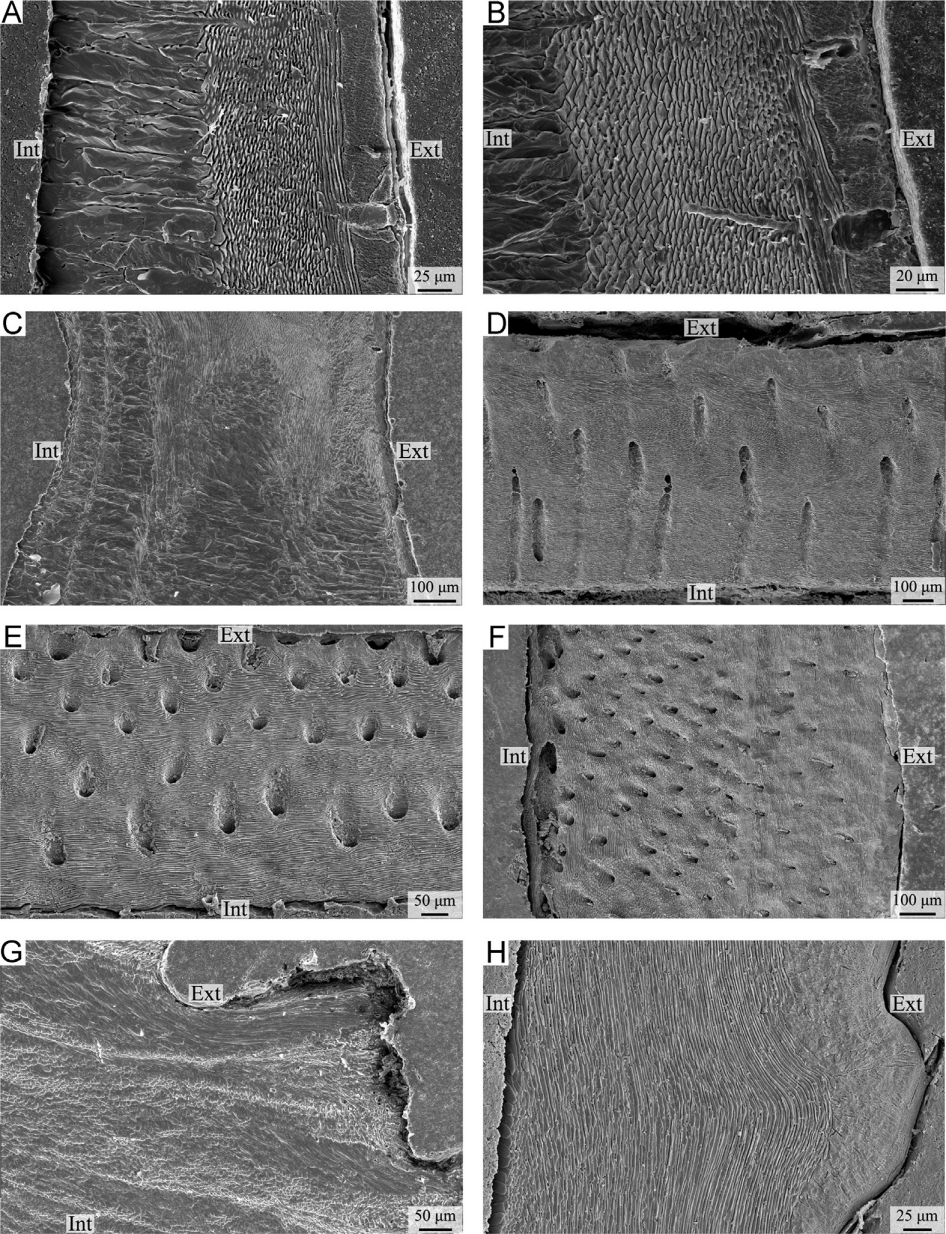
A-C) *Gryphus vitreus*. A-B) complete shell succession showing the change in the orientation of fibres from oblique to transverse from the exterior to the interior of the secondary layer (A: anterior part, ventral valve, longitudinal section; B: central part, dorsal valve, longitudinal section); C) complete shell succession showing the change in the orientation of fibres from transverse to oblique from the exterior to the interior of the secondary layer, and the alternations of the secondary and tertiary layers (posterior part, ventral valve, longitudinal section). D-F) *Magasella sanguinea*. Complete shell succession showing several sublayers with variable fibre orientation (D: anterior part, ventral valve, longitudinal section; E: central part, dorsal valve, longitudinal section; F: posterior part, ventral valve, longitudinal section); G-H) *Notosaria nigricans*. G) secondary layer showing several sublayers with variable fibre orientation (anterior part, ventral valve, longitudinal section); H) complete shell succession showing longitudinal to oblique fibres, except for a few transversally oriented fibres in the internal part (posterior part, ventral valve, longitudinal section). Ext: external part of the shell; Int: internal part of the shell.

We followed SEM sample preparation as suggested by Crippa et al. [3]. The specimens were embedded in a transparent bicomponent epoxy resin and cut along the longitudinal (or transversal) axis using a low speed saw with a thin diamond blade. To remove the organic matter within the shell, samples were immersed in 36 volume hydrogen peroxide (H_2_O_2_) for 24 h. Sectioned surfaces were smoothed with silicon carbide (SiC) powder of two different granulometries (400 and 1000 grit sizes), etched with 5% hydrochloric acid (HCl) for 3 s, and rinsed in deionised water and dried. They were gold-coated and observed by Cambridge S-360 scanning electron microscope with a lanthanum hexaboride (LaB_6_) cathodes and operating at an acceleration voltage of 20 kV at Dipartimento di Scienze della Terra “A. Desio”, University of Milan, Italy. Plate 1, Plate 2, Plate 3, Plate 4 show the shell microstructure of the six brachiopod species analyzed: *Liothyrella uva, Gryphus vitreus, Liothyrella neozelanica, Calloria incospicua, Magasella sanguinea* and *Notosaria nigricans.*

### 2.3 EBSD

**Plate 5:**
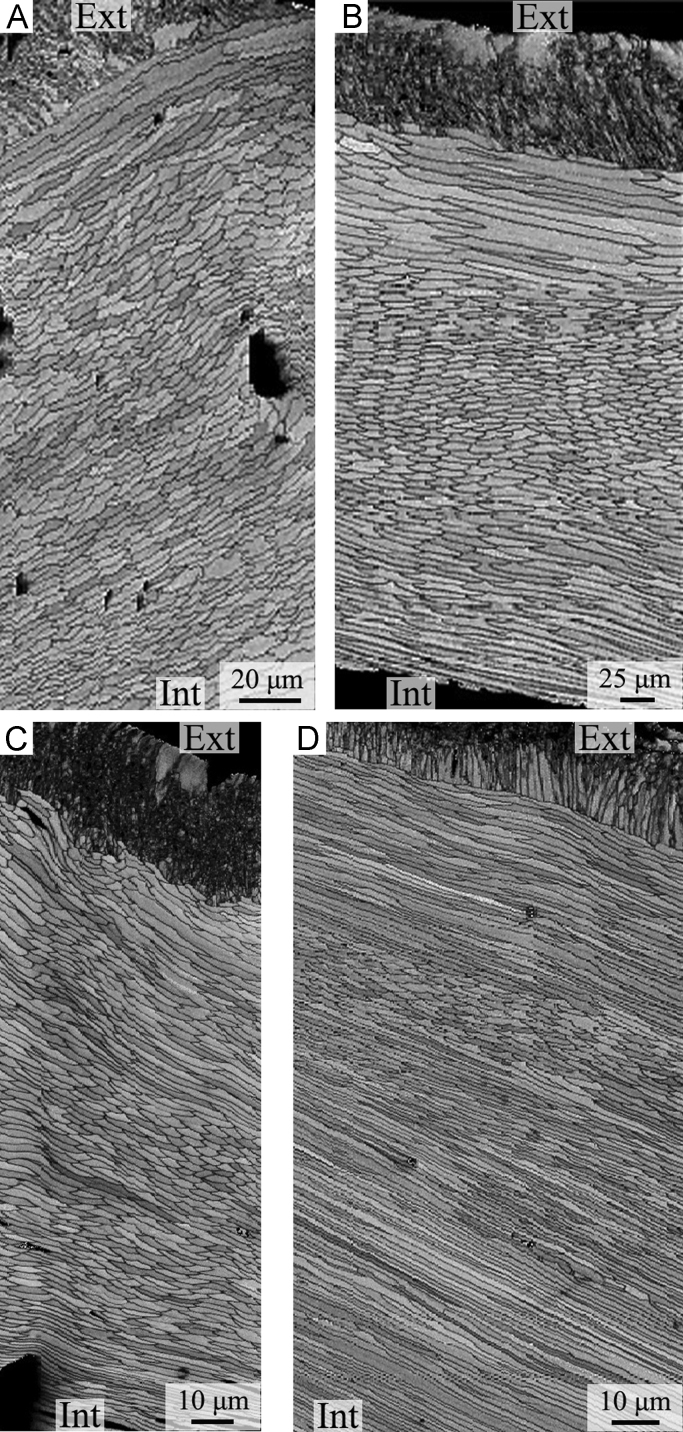
EBSD band contrast images visualizing the difference in microstructure of two layer brachiopod shells that comprise the primary and the fibrous shell layers. (A: *Liothyrella uva*; B: *Calloria inconspicua*; C: *Magasella sanguinea*; D: *Notosaria nigricans*). Ext: external part of the shell; Int: internal part of the shell.

For EBSD measurements brachiopod shells were embedded in epoxy resin and were cut along and perpendicular to the median plane of the investigated shells. Surfaces of the embedded specimens were subjected to several sequential mechanical grinding and polishing steps down to a grain size of 1 μm. The final polishing step was carried out with colloidal alumina (particle size ~ 0.06 μm) in a vibratory polisher. Sample surfaces were coated with 4–6 nm of carbon. EBSD measurements were carried out at the Department of Earth and Environmental Sciences, LMU Munich, Munich, Germany, on a Hitachi SU5000 field emission SEM, equipped with a Nordlys II EBSD detector and AZTec acquisition software. The SEM was operated at 15 and 20 kV; measurements were evaluated with CHANNEL 5 HKL software [11], [12]. EBSD data are presented as band contrast measurement images, a grey scale component that gives the signal strength of the EBSD Kikuchi diffraction pattern in each measurement point. Accordingly, the strength of the diffraction signal is high when a mineral is detected whereas it is weak or absent when a polymer is scanned. A high diffraction signal is shown with light, while a weak signal is visualized with dark grey colors in the band contrast measurement image. Plate 5 shows EBSD band contrast measurement images of two layer shells (*L. uva, C. incospicua, M. sanguinea, N. nigricans*).

### 2.4 Statistical analyses

Based on SEM micrographs, each fibre, with regular and symmetrical cross sectional outline, was chosen for morphometric measurements (1197 measurements) from different ontogenetic stages; fibres were first outlined using Adobe Photoshop CS6, and then all parameters (e.g. Max Feret diameter, Min Feret diameter, Area, Perimeter, Convex area and Convex perimeter) were measured by Image-Pro Plus 6.0 and ImageJ.

**Fig. 1:**
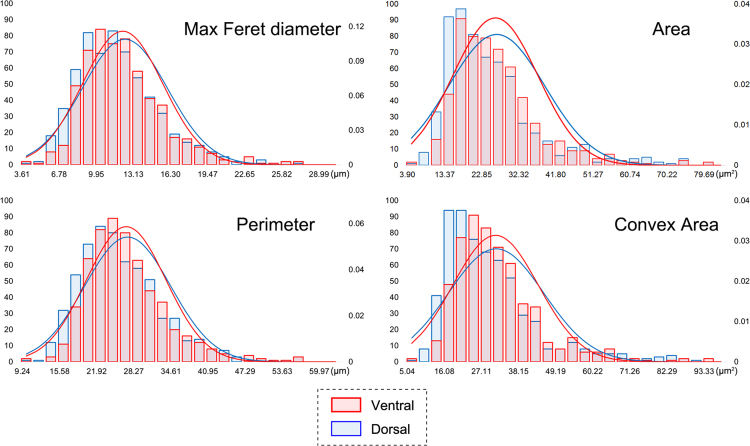
Distribution plots of the original parameters of all six species in the ventral valve (red) and dorsal valve (blue). (For interpretation of the references to color in this figure legend, the reader is referred to the web version of this article.)

**Fig. 2:**
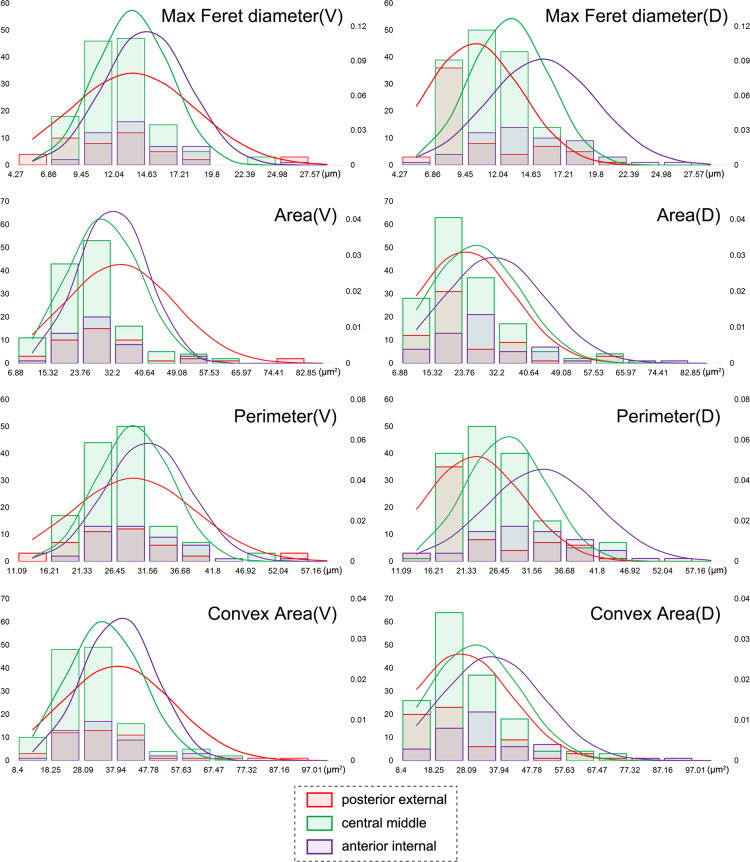
Distribution plots of the original parameters from different positions in ontogenetic direction (red: posterior external; green: central middle; violet: anterior internal; V: ventral; D: dorsal). (For interpretation of the references to color in this figure legend, the reader is referred to the web version of this article.)

**Fig. 3:**
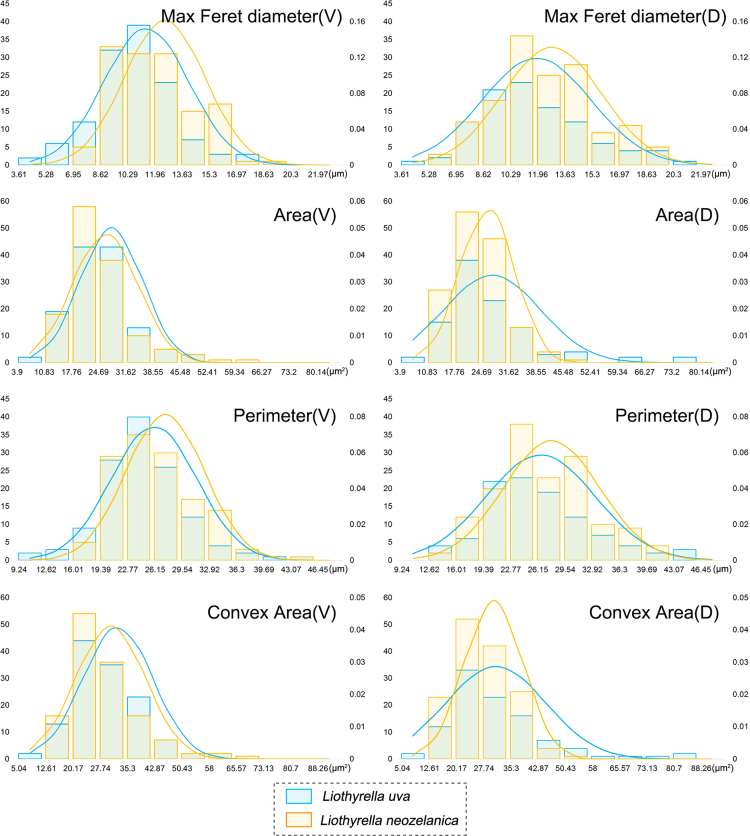
Distribution plots of the original parameters of *Liothyrella uva* (light blue) and *Liothyrella neozelanica* (orange) (V: ventral; D: dorsal). (For interpretation of the references to color in this figure legend, the reader is referred to the web version of this article.)

The frequency distribution plots of the most significant parameters (Area, Perimeter, Max Feret diameter, Convex Area) were calculated and drawn by Excel 2013 (FREQUENCY function and NORM.DIST function) (Fig. 1, Fig. 2, Fig. 3) [cf. 13].

**Fig. 4:**
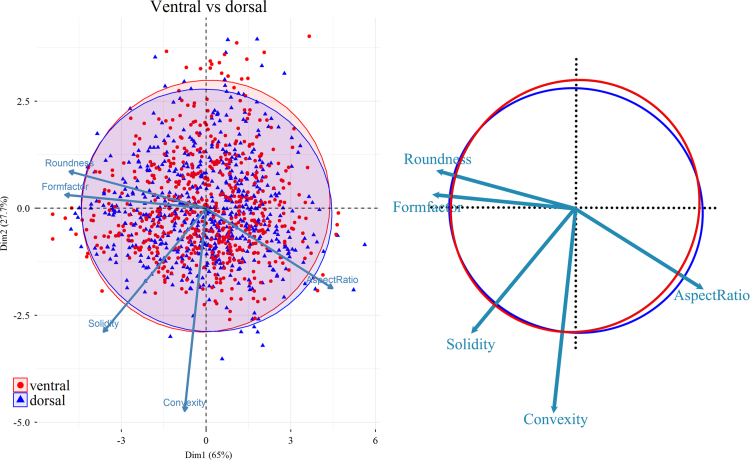
PCA (Principal Component Analysis) plot of fibres from ventral (red) and dorsal (blue) valves. Five variables (Roundness, Formfactor, Solidity, Convexity, AspectRatio) are considered for the PCA; the longer the arrow, the greater the correlation between the specific factor and that direction in the PCA space. 95% confidence ellipse and centroids (larger symbols, overlapping in the central point in this case) for each data sets are also shown in the plot. (For interpretation of the references to color in this figure legend, the reader is referred to the web version of this article.)

**Fig. 5:**
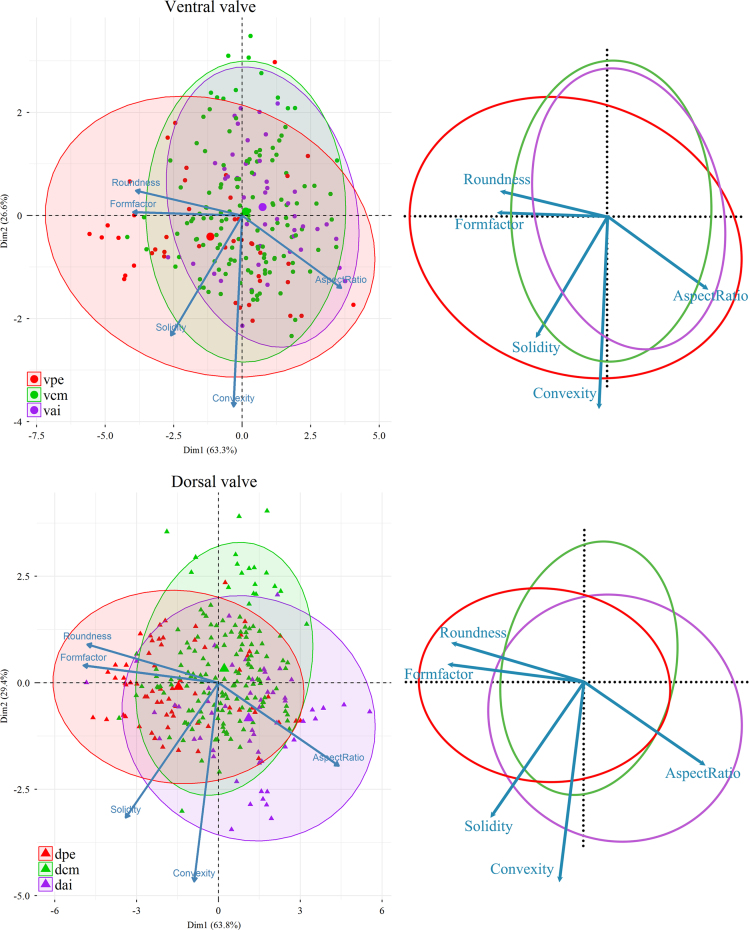
PCA (Principal Component Analysis) plots showing the morphological change of the fibres in the ontogenetic direction. Five variables (Roundness, Formfactor, Solidity, Convexity, AspectRatio) are considered for the PCA; the longer the arrow, the greater the correlation between the specific factor and that direction in the PCA space (vpe: ventral posterior external; vcm: ventral central middle; vai: ventral anterior internal; dpe: dorsal posterior external; dcm: dorsal central middle; dai: dorsal anterior internal). 95% confidence ellipse and centroids (larger symbols) for each data groups are also shown in the plot.

**Fig. 6:**
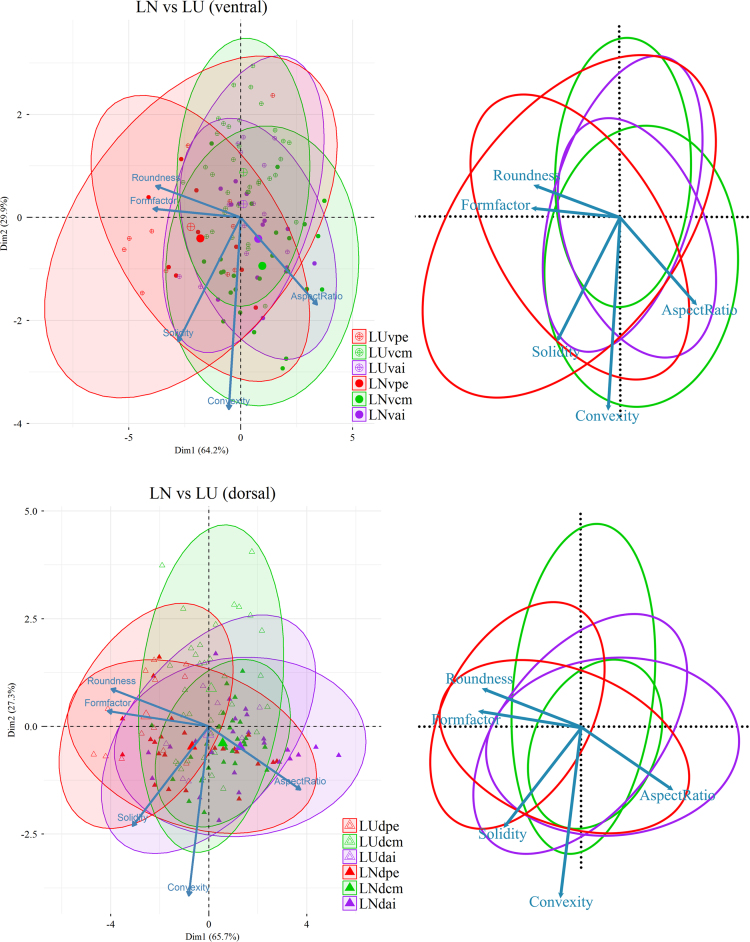
PCA (Principal Component Analysis) plots showing the comparison of the fibres between *Liothyrella uva* and *Liothyrella neozelanica*. Five variables (Roundness, Formfactor, Solidity, Convexity, AspectRatio) are considered for the PCA; the longer the arrow, the greater the correlation between the specific factor and that direction in the PCA space. (LUvpe: *Liothyrella uva* ventral posterior external; LUvcm: *Liothyrella uva* ventral central middle; LUvai: *Liothyrella uva* ventral anterior internal; LNvpe: *Liothyrella neozelanica* ventral posterior external; LNvcm: *Liothyrella neozelanica* ventral central middle; LNvai: *Liothyrella neozelanica* ventral anterior internal; LUdpe: *Liothyrella uva* dorsal posterior external; LUdcm: *Liothyrella uva* dorsal central middle; LUdai: *Liothyrella uva* dorsal anterior internal; LNdpe: *Liothyrella neozelanica* dorsal posterior external; LNdcm: *Liothyrella neozelanica* dorsal central middle; LNdai: *Liothyrella neozelanica* dorsal anterior internal). 95% confidence ellipse and centroids (larger symbols) for each data sets are also shown in the plot.

Based on the six measured parameters, five shape descriptors were calculated: Formfactor (circularity, 4π × Area/Perimeter^2^), Roundness (4Area/π × Max Feret diameter^2^), Aspect Ratio (Max Feret diameter/Min Feret diameter), Convexity (Convex Perimeter/Perimeter), and Solidity (Area/Convex Area) [14]. For data visualization and dimension reduction, principal component analysis (PCA) was performed on the five shape descriptors using R 3.3.0 (Fig. 4, Fig. 5, Fig. 6) [2]. We used the function *prcomp* for principal component analysis and *fviz_pca_biplot* for plot; the biplots were created using the package *factoextra* [15].

Independent-sample *t*-tests were performed using SPSS Statistics (IBM Version 22.0. Armonk, NY) (Table 2, Table 3, Table 4, Table 5, Table 6, Table 7, Table 8, Table 9). A *p*-value ≤ 0.05 is considered significant.

**Table 2:** *T*-test of fibres size and shape data of the ventral valve vs the dorsal valve (LU: *Liothyrella uva*; GV: *Gryphus vitreus*; LN: *Liothyrella neozelanica*; CI: *Calloria incospicua*; MS: *Magasella sanguinea*; NN: *Notosaria nigricans*). Significant values (*p*-value ≤0.05) are marked in bold style.

	Area	Perimeter	Max Feret diameter	Roundness	Convexity
LU	t(165.165)=1.429, *p*=0.155	t(188.750)=1.984, ***p*****=0.049**	t(187.755)=2.392, ***p*****=0.018**	t(228)=−2.632, ***p*****=0.009**	t(228)=1.130, *p*=0.260
GV	t(275)=−7.376, ***p*****<0.001**	t(233.644)=−5.890, ***p*****<0.001**	t(234.192)=−5.414, ***p*****<0.001**	t(275)=0.947, *p*=0.344	t(275)=−2.784, ***p*****=0.006**
LN	t(279)=−1.099, *p*=0.273	t(276.009)=1.054, *p*=0.293	t(275.477)=1.511, *p*=0.132	t(263.010)=−2.479, ***p*****=0.014**	t(279)=0.120, *p*=0.905
CI	t(68)=−2.509, ***p*****=0.015**	t(68)=−3.564, ***p*****=0.001**	t(68)=−3.394, ***p*****=0.001**	t(41.294)=2.727, ***p*****=0.009**	t(68)=1.412, *p*=0.163
MS	t(133)=−0.723, *p*=0.471	t(133)=−0.834, *p*=0.406	t(133)=−0.737, *p*=0.463	t(133)=−0.029, *p*=0.977	t(133)=0.005, *p*=0.996
NN	t(202)=1.951, *p*=0.052	t(202)=−0.055, *p*=0.956	t(202)=−0.583, *p*=0.561	t(178.721)=3.866, ***p*****<0.001**	t(202)=−1.506, *p*=0.134
All 6 species	t(1195)=−2.340, ***p*****=0.019**	t(1194.446)=−1.970, ***p*****=0.049**	t(1195)=−1.574, *p*=0.116	t(1195)=−0.723, *p*=0.470	t(1195)=−0.165, *p*=0.869

**Table 3:** *T*-test of fibres size and shape data of the ventral valve vs the dorsal valve in different positions of the shell (pe: posterior external; cm: central middle; ai: anterior internal). Significant values (*p*-value ≤ 0.05) are marked in bold style.

Position	Area	Perimeter	Max Feret diameter	Roundness	Convexity
pe	t(106)=−2.649, ***p*****=0.009**	t(106)=−2.587, ***p*****=0.011**	t(106)=−2.423, ***p*****=0.017**	t(72.163)=0.279, *p*=0.781	t(106)=−1.991, ***p*****=0.049**
cm	t(290)=−1.210, *p*=0.227	t(290)=−1.413, *p*=0.159	t(290)=−1.312, *p*=0.191	t(290)=−0.467, *p*=0.641	t(290)=−2.437, ***p*****=0.015**
ai	t(98)=0.032, *p*=0.974	t(98)=0.654, *p*=0.515	t(98)=0.970, *p*=0.334	t(98)=−1.297, *p*=0.198	t(98)=3.233, ***p*****=0.002**

**Table 4:** *T*-test of fibres size and shape data of the anterior internal vs central middle vs posterior external parts of both the ventral valve (vpe, vcm, vai) and the dorsal valve (dpe, dcm, dai), considering all the six analyzed species together. See caption of Fig. 5 for the legend. Significant values (*p*-value ≤ 0.05) are marked in bold style.

Valve and position	Area	Perimeter	Max Feret diameter	Roundness	Convexity
Vpe vs Vcm	t(56.715)=−2.192, ***p*****=0.033**	t(53.925)=−0.505, *p*=0.615	t(53.307)=−0.241, *p*=0.811	t(50.796)=−3.335, ***p*****=0.002**	t(176)=−2.854, ***p*****=0.005**
Vpe vs Vai	t(87)=1.136, *p*=0.259	t(87)=−1.126, *p*=0.263	t(87)=−1.325, *p*=0.188	t(57.287)=4.468, ***p*****<0.001**	t(87)=2.884, ***p*****=0.005**
Vcm vs Vai	t(177)=−1.340, *p*=0.182	t(177)=−2.623, ***p*****=0.009**	t(177)=−2.619, ***p*****=0.010**	t(177)=2.394, ***p*****=0.018**	t(177)=0.822, *p*=0.412
Dpe vs Dcm	t(220)=−0.153, *p*=0.878	t(100.527)=−2.322, ***p*****=0.022**	t(99.878)=−2.598, ***p*****=0.011**	t(83.739)=6.264, ***p*****<0.001**	t(152.038)=3.566, ***p*****<0.001**
Dpe vs Dai	t(117)=−1.733, *p*=0.086	t(117)=−4.889, ***p*****<0.001**	t(117)=−5.402, ***p*****<0.001**	t(116.994)=7.581, ***p*****<0.001**	t(117)=−2.241, ***p*****=0.027**
Dcm vs Dai	t(211)=−1.992, ***p*****=0.048**	t(75.180)=−3.762, ***p*****<0.001**	t(74.481)=−4.138, ***p*****<0.001**	t(211)=4.108, ***p*****<0.001**	t(211)=−5.119, ***p*****<0.001**

**Table 5:** *T*-test of fibres size and shape data in different positions of the ventral valve. See captions of Fig. 5 and Table 2 for the legend. Significant values (*p*-value ≤ 0.05) are marked in bold style.

Species and position	Area	Perimeter	Max Feret diameter	Roundness	Convexity
LUvpe vs LUvai	t(22)=0.079, *p*=0.938	t(17.461)=−1.132, *p*=0.273	t(16.910)=−1.314, *p*=0.206	t(12.538)=3.013, ***p*****=0.010**	t(22)=1.284, *p*=0.213
GVvpe vs GVvai	t(15)=2.502, ***p*****=0.024**	t(15)=0.680, *p*=0.507	t(15)=0.355, *p*=0.727	t(15)=1.158, *p*=0.265	t(15)=0.779, *p*=0.448
LNvpe vs LNvai	t(21)=1.193, *p*=0.246	t(21)=3.551, ***p*****=0.002**	t(21)=3.758, ***p*****=0.001**	t(21)=−3.726, ***p*****=0.001**	t(21)=−0.715, *p*=0.482
CIvpe vs CIvai	–	–	t(1.293)=0.657, *p*=0.609	t(1.087)=−5.131, *p*=0.108	t(1.481)=2.815, *p*=0.147
MSvpe vs MSvai	t(2.081)=−1.538, *p*=0.259	t(4)=−16.618, ***p*****<0.001**	t(4)=−15.308, ***p*****<0.001**	t(4)=6.087, ***p*****=0.002**	t(4)=1.527, *p*=0.202
NNvpe vs NNvai	t(13)=2.409, ***p*****=0.032**	t(13) =1.517, *p*=0.153	t(13)=1.445, *p*=0.172	t(13)=0.561, *p*=0.574	t(13)=0.877, *p*=0.396

**Table 6:** *T*-test of fibres size and shape data in different positions of the dorsal valve. See caption of Fig. 5 and Table 2 for the legend. Significant values (*p*-value ≤ 0.05) are marked in bold style.

Species and position	Area	Perimeter	Max Feret diameter	Roundness	Convexity
LUdpe vs LUdai	t(6.673)=−1.127, *p*=0.299	t(6.548)=−1.966, *p*=0.093	t(6.766)=−2.314, *p*=0.055	t(18)=4.340, ***p*****<0.001**	t(18)=0.100, *p*=0.921
GVdpe vs GVdai	t(12.345)=5.286, ***p*****<0.001**	t(11.772)=−8.424, ***p*****<0.001**	t(11.897)=−9.113, ***p*****<0.001**	t(21.023)=10.459, ***p*****<0.001**	t(26)=−4.931, ***p*****<0.001**
LNdpe vs LNdai	t(40.052)=−0.794, *p*=0.432	t(37.697)=−2.353, ***p*****=0.024**	t(37.929)=−2.384, ***p*****=0.022**	t(40.869)=3.232, ***p*****=0.002**	t(45)=0.208, *p*=0.836
NNdpe vs NNdai	t(16)=0.396, *p*=0.697	t(16)=−0.801, *p*=0.435	t(16)=−1.075, *p*=0.298	t(16)=1.773, *p*=0.088	t(16)=−2.280, ***p*****=0.037**

**Table 7:** *T*-test of fibres size and shape data of Group1-three layer shells (*Gryphus vitreus* and *Liothyrella neozelanica*) vs Group 2-two layer shells (*Liothyrella uva, Calloria inconspicua, Magasella sanguinea* and *Notosaria nigricans*) for different positions of the ventral valve and dorsal valve. See caption of Fig. 5 for the legend. Significant values (*p*-value ≤ 0.05) are marked in bold style.

Group and position	Area	Perimeter	Max Feret diameter	Roundness	Convexity
Gr.1 vpe vs Gr.2 vpe	t(27.938)=−0.622, *p*=0.539	t(27.378)=−0.605, *p*=0.549	t(28.153)=−0.493, *p*=0.626	t(36.757)=−0.748, *p*=0.460	t(42)=1.136, *p*=0.262
Gr.1vcm vs Gr.2vcm	t(132)=−2.350, ***p*****=0.020**	t(128.900)=−0.653, *p*=0.515	t(131.623)=0.032, *p*=0.975	t(119.932)=−4.417, ***p*****<0.001**	t(118.499)=1.586, *p*=0.115
Gr.1vai vs Gr.2vai	t(39.475)=−0.795, *p*=0.432	t(40.287)=−0.848, *p*=0.402	t(40.571)=−0.667, *p*=0.509	t(43)=−0.033, *p*=0.974	t(43)=1.136, *p*=0.262
Gr.1dpe vs Gr.2dpe	t(33.052)=−2.994, ***p*****=0.005**	t(62)=−1.644, *p*=0.105	t(62)=−1.130, *p*=0.263	t(62)=−1.702, *p*=0.094	t(34.514)=1.292, *p*=0.205
Gr.1dcm vs Gr.2dcm	t(130.484)=−5.613, ***p*****<0.001**	t(155.250)=−3.537, ***p*****=0.001**	t(155.766)=−2.897, ***p*****=0.004**	t(156)=−3.230, ***p*****=0.002**	t(156)=−0.066, *p*=0.947
Gr.1dai vs Gr.2dai	t(21.387)=−0.692, *p*=0.496	t(22.352)=0.456, *p*=0.653	t(22.757)=0.631, *p*=0.534	t(53)=−2.341, ***p*****=0.023**	t(53)=1.833, *p*=0.072
Gr.1v vs Gr.2v	t(578.998)=−3.254, ***p*****=0.001**	t(576.984)=−1.133, *p*=0.258	t(577.130)=−0.334, *p*=0.738	t(579)=−3.475, ***p*****=0.001**	t(567.776)=5.464, ***p*****<0.001**
Gr.1d vs Gr.2d	t(395.017)=−8.935, ***p*****<0.001**	t(509.357)=−4.129, ***p*****<0.001**	t(519.510)=−2.881, ***p*****=0.004**	t(560.685)=−6.134, ***p*****<0.001**	t(571.282)=2.838, ***p*****=0.005**

**Table 8:** *T*-test of fibres size and shape data of *Liothyrella neozelanica* vs *Gryphus vitreus* (both three-layer shells) for different positions in the ventral valve and dorsal valve. See captions of Fig. 5 and Table 2 for the legend. Significant values (*p*-value ≤0.05) are marked in bold style.

Species and position	Area	Perimeter	Max Feret diameter	Roundness	Convexity
LNvpe vs GVvpe	t(20)=3.222, ***p*****=0.004**	t(20)=3.961, ***p*****=0.001**	t(20)=3.806, ***p*****=0.001**	t(20)=−1.727, *p*=0.100	t(20)=3.586, ***p*****=0.002**
LNvcm vs GVvcm	t(45)=0.273, *p*=0.786	t(45)=0.069, *p*=0.945	t(45)=0.018, *p*=0.986	t(42.265)=0.529, *p*=0.600	t(45)=−1.375, *p*=0.176
LNvai vs GVvai	t(16)=−0.714, *p*=0.486	t(16)=−0.412, *p*=0.686	t(16)=−0.211, *p*=0.836	t(16)=−0.456, *p*=0.654	t(16)=2.580, ***p*****=0.020**
LNdpe vs GVdpe	t(27.016)=−3.609, ***p*****=0.001**	t(23.790)=−4.157, ***p*****<0.001**	t(23.940)=−4.275, ***p*****<0.001**	t(37)=3.441, ***p*****=0.001**	t(37)=−0.939, *p*=0.354
LNdcm vs GVdcm	t(35.615)=−5.782, ***p*****<0.001**	t(36.280)=−5.303, ***p*****<0.001**	t(37.699)=−5.524, ***p*****<0.001**	t(65)=2.686, ***p*****=0.009**	t(62.375)=−4.495, ***p*****<0.001**
LNdai vs GVdai	t(34)=2.023, *p*=0.051	t(34)=1.910, *p*=0.065	t(34)=2.160, ***p*****=0.038**	t(33.054)=−1.639, *p*=0.111	t(34)=3.929, ***p*****<0.001**
LNv vs GVv	t(225)=1.215, *p*=0.225	t(225)=1.657, *p*=0.099	t(225)=1.804, *p*=0.073	t(217.032)=−1.385, *p*=0.167	t(225)=0.634, *p*=0.527
LNd vs GVd	t(329)=−5.660, ***p*****<0.001**	t(329)=−5.107, ***p*****<0.001**	t(329)=−4.979, ***p*****<0.001**	t(329)=2.180, ***p*****=0.030**	t(323.389)=−2.998, ***p*****=0.003**

**Table 9:** *T*-test of fibres size and shape data of Group NZ New Zealand (*Calloria inconspicua, Magasella sanguinea* and *Notosaria nigricans*) vs Group LN New Zealand (*Liothyrella neozelanica*) vs Group MED Mediterranean (*Gryphus vitreus*) vs Group ANT Antarctica (*Liothyrella uva*); (v: ventral valve; d: dorsal valve). Significant values (*p*-value ≤ 0.05) are marked in bold style.

Group and position	Area	Perimeter	Max Feret diameter	Roundness	Convexity
Gr.NZv vs Gr.LNv	t(357.973)=4.452, ***p*****<0.001**	t(357.548)=3.611, ***p*****<0.001**	t(357.515)=3.327, ***p*****=0.001**	t(358)=0.237, *p*=0.814	t(330.310)=−1.943, *p*=0.053
Gr.NZv vs Gr.MEDv	t(298.514)=3.268, ***p*****=0.001**	t(302.183)=2.070, ***p*****=0.039**	t(300.104)=1.647, *p*=0.101	t(207.223)=1.775, *p*=0.077	t(317)=−2.147, ***p*****=0.033**
Gr.NZv vs Gr.ANTv	t(351.958)=4.620, ***p*****<0.001**	t(349.047)=5.771, ***p*****<0.001**	t(350.600)=6.487, ***p*****<0.001**	t(352)=−4.981, ***p*****<0.001**	t(233.672)=5.068, ***p*****<0.001**
Gr.LNv vs Gr.MEDv	t(225)=−1.215, *p*=0.215	t(225)=−1.657, *p*=0.099	t(225)=−1.804, *p*=0.073	t(217.032)=1.385, *p*=0.167	t(225)=−0.634, *p*=0.527
Gr.LNv vs Gr.ANTv	t(260)=0.154, *p*=0.878	t(260)=2.699, ***p*****=0.007**	t(260)=3.833, ***p*****<0.001**	t(260)=−4.797, ***p*****<0.001**	t(22.742)=6.538, ***p*****<0.001**
Gr.MEDv vs Gr.ANTv	t(219)=1.387, *p*=0.167	t(219)=4.077, ***p*****<0.001**	t(219)=5.299, ***p*****<0.001**	t(219)=−6.141, ***p*****<0.001**	t(218.557)=6.382, ***p*****<0.001**
Gr.NZd vs Gr.LNd	t(258.275)=6.246, ***p*****<0.001**	t(315.809)=1.691, *p*=0.092	t(318.466)=0.705, *p*=0.481	t(326.954)=5.898, ***p*****<0.001**	t(327.455)=−2.565, ***p*****=0.011**
Gr.NZd vs Gr.MEDd	t(246.940)=9.713, ***p*****<0.001**	t(308.306)=5.924, ***p*****<0.001**	t(314.858)=4.873, ***p*****<0.001**	t(348.395)=4.027, ***p*****<0.001**	t(365)=0.543, *p*=0.587
Gr.NZd vs Gr.ANTd	t(256.290)=3.165, ***p*****=0.002**	t(260.731)=2.287, ***p*****=0.023**	t(261.222)=2.186, ***p*****=0.030**	t(252.246)=0.944, *p*=0.346	t(174.498)=3.192, ***p*****=0.002**
Gr.LNd vs Gr.MEDd	t(329)=5.660, ***p*****<0.001**	t(329)=5.107, ***p*****<0.001**	t(329)=4.979, ***p*****<0.001**	t(329)=−2.180, ***p*****=0.030**	t(323.389)=2.998, ***p*****=0.003**
Gr.LNd vs Gr.ANTd	t(145.357)=−2.122, ***p*****=0.035**	t(247)=0.939, *p*=0.349	t(247)=1.800, *p*=0.073	t(247)=−4.743, ***p*****<0.001**	t(154.474)=5.114, ***p*****<0.001**
Gr.MEDd vs Gr.ANTd	t(137.337)=−5.444, ***p*****<0.001**	t(284)=−3.360, ***p*****=0.001**	t(284)=−2.395, ***p*****=0.017**	t(284)=−2.849, ***p*****=0.005**	t(284)=2.792, ***p*****=0.006**

## Funding

This project has received funding from the European Union's Horizon 2020 research and innovation programme under grant agreement No 643084.
