# Supplementary material for: Microstructural data of six recent brachiopod species: SEM, EBSD, morphometric and statistical analyses
Source: Data Brief. 2018 Mar 6;18:300–18. doi: 10.1016/j.dib.2018.02.071 (PMC5995785; doi:10.1016/j.dib.2018.02.071)
Supplement: Supplementary file 1 — Supplementary material. [file mmc1.docx]

**Declaration of interest** DIB-D-17-01209

“Microstructural data of six recent brachiopod species: SEM, EBSD, morphometric and statistical analyses”

Authored by

Facheng Ye, Gaia Crippa, Claudio Garbelli, Erika Griesshaber

‘Declarations of interest: none’

We wish to confirm that there are no known conflicts of interest associated with this publication and there has been no significant financial support for this work that could have influenced its outcome.

Kind regards,

Facheng Ye
